# Predictive Value of Left Atrial Strain Combined with Low-Voltage Substrate for Recurrence After Radiofrequency Catheter Ablation in Persistent Atrial Fibrillation

**DOI:** 10.3390/diagnostics16203303

**Published:** 2026-10-11

**Authors:** Na Ma, Xiaoran Cui, Ruibin Li, Yichen Li, Yan Wang, Jidong Zhang

**Affiliations:** Department of Cardiology, The Second Hospital of Hebei Medical University, Shijiazhuang 050000, China

**Keywords:** persistent atrial fibrillation, radiofrequency catheter ablation, left atrial strain, low-voltage area, recurrence prediction

## Abstract

**Background/Objectives:** To evaluate associations of left atrial strain and low-voltage area (LVA) with recurrence after radiofrequency catheter ablation (RFCA) for persistent atrial fibrillation. **Methods:** This retrospective cohort included 66 patients undergoing first-time RFCA. Strain was measured before ablation and at 3 months, and voltage mapping quantified LVA. Recurrence after the 3-month blanking period was assessed during approximately 3 months of intermittent follow-up. **Results:** Recurrence was detected in 16 patients (24.2%). Preoperative left atrial reservoir strain (LASr) and contraction strain were lower in patients with recurrence. At 3 months, LASr-3mo and contraction strain remained lower, whereas LVA percentage was higher (11.50% vs. 3.00%; *p* < 0.001). LASr-3mo correlated negatively with LVA (r = −0.345; *p* = 0.005). In multivariable analyses, LASr-3mo (OR = 0.617; *p* < 0.001) and Delta LASr (OR = 0.461; *p* < 0.001) were inversely associated with recurrence, whereas LVA was positively associated (OR = 1.069; *p* = 0.044). The LASr-3mo plus LVA model showed promising apparent discrimination (apparent AUC = 0.923; bootstrap-corrected AUC = 0.913). **Conclusions:** LASr-3mo may contribute to postoperative risk reassessment. LVA remains an exploratory observational marker because voltage mapping influenced treatment selection. The cohort-derived 4.5% LVA cut-off and model performance require prospective external validation using standardized ablation protocols.

## 1. Introduction

Atrial fibrillation (AF) is one of the most common arrhythmias encountered in clinical practice, and its prevalence increases markedly with age. According to the China Cardiovascular Health and Disease Report 2024, approximately 4.87 million people in China have AF, and this number continues to increase with population ageing [1]. The Global Burden of Disease Study reported that the global number of patients with AF exceeds 59 million, nearly three times the estimate in 1990, and is projected to exceed 100 million by 2050 [2]. AF causes palpitations, fatigue, and reduced exercise tolerance and substantially increases the risks of heart failure, ischemic stroke, and all-cause mortality. AF-related strokes account for 20–25% of ischemic strokes and are associated with high disability and mortality [3,4].

Radiofrequency catheter ablation (RFCA) is a first-line rhythm-control strategy for symptomatic AF, particularly in patients who are refractory to or intolerant of antiarrhythmic drugs [5,6]. The 2024 European Society of Cardiology guidelines and the American Heart Association/American College of Cardiology/Heart Rhythm Society guidelines recommend catheter ablation as a Class I indication for maintaining sinus rhythm in appropriately selected symptomatic patients [7,8]. Nevertheless, despite advances in ablation technology, recurrence after ablation remains common in persistent atrial fibrillation (PeAF), with reported rates of 30–50%, which are higher than those observed in paroxysmal AF [9,10]. Early identification of patients at high risk of recurrence is therefore central to individualized AF management.

The mechanisms underlying AF recurrence are closely related to atrial remodeling, including electrical, structural, and functional remodeling [11,12]. Electrical remodeling is characterized by shortening of the atrial effective refractory period, slowing of conduction velocity, and formation of re-entry circuits. Structural remodeling includes atrial fibrosis, cardiomyocyte hypertrophy and apoptosis, and extracellular matrix deposition. Functional remodeling manifests as impaired atrial contraction and relaxation [13]. Patients with PeAF usually have longer AF duration, more advanced atrial remodeling, and a more diseased atrial substrate, which may explain the high recurrence rate after ablation [14]. Reliable markers that quantify atrial substrate abnormalities are therefore needed to guide ablation strategies and predict recurrence.

During electroanatomical mapping (EAM), low-voltage area (LVA) can serve as a surrogate marker of atrial fibrosis and provides real-time visualization during ablation [15]. LVA is commonly defined as a bipolar voltage amplitude < 0.5 mV in sinus rhythm or <0.25 mV during AF, corresponding pathologically to fibrosis or scar tissue [16,17]. Larger LVA burden has been associated with a higher risk of recurrence after AF ablation [18]. However, LVA assessment has limitations: it primarily reflects static structural abnormalities rather than dynamic functional status; mapping accuracy is influenced by operator experience, catheter contact, filter settings, and other procedural factors; and voltage thresholds, mapping density, and rhythm during mapping differ across studies [19,20]. As a single marker, LVA therefore has only moderate predictive performance.

The left atrium has a key role in the development and maintenance of AF. Left atrial enlargement and dysfunction are not only consequences of AF but also contributors to its persistence and recurrence. Common clinical measures of left atrial structure, such as left atrial diameter (LAD) and left atrial volume index (LAVI), are simple and reproducible but mainly reflect static anatomical remodeling. They may lag behind myocardial fibrosis and functional impairment and may miss patients with early functional abnormalities despite limited chamber enlargement [21]. More sensitive measures of left atrial function are therefore needed for early substrate assessment and risk prediction.

Left atrial strain (LAS) derived from two-dimensional speckle-tracking echocardiography (2D-STE) has emerged as a sensitive marker of atrial functional remodeling [22,23]. LAS includes left atrial reservoir strain (LASr), conduit strain (LAScd), and contraction strain (LASct), which reflect reservoir, conduit, and pump function, respectively [22,24]. Reduced LAS has been associated with atrial fibrosis and has been shown to predict recurrence after ablation [25]. However, most previous studies focused on preoperative baseline values and paid less attention to dynamic postoperative recovery. Although LAS and LVA both reflect atrial substrate abnormalities, they capture different dimensions: LVA reflects structural and electrical fibrosis, whereas LAS reflects mechanical deformation. Combining these markers may provide a dual structure-function assessment and improve recurrence prediction, particularly in patients with PeAF [26].

This study aimed to evaluate the association between left atrial strain and low-voltage substrate in patients with PeAF, examine the apparent predictive performance of LAS combined with LVA percentage for recurrence after RFCA, and explore the performance of LAS-based models across LVA subgroups.

## 2. Methods

### 2.1. Study Design and Participants

This retrospective cohort study enrolled patients with PeAF who were admitted to the Department of Cardiology, the Second Hospital of Hebei Medical University, between 1 May 2024 and 31 January 2025 and underwent first-time RFCA. After application of the inclusion and exclusion criteria, 66 patients were included. The inclusion criteria were as follows: (1) age 18–80 years; (2) diagnosis of PeAF according to the 2024 ESC Guidelines for the Management of Atrial Fibrillation [8]; (3) first-time RFCA; (4) preoperative transesophageal echocardiography confirming the absence of left atrial thrombus; (5) completed intraoperative high-density left atrial voltage mapping; and (6) regular postoperative follow-up for at least 6 months.

The exclusion criteria were as follows: (1) previous RFCA or surgical ablation; (2) congenital heart disease, idiopathic cardiomyopathy (hypertrophic, dilated, or restrictive), severe valvular heart disease, severe hepatic or renal insufficiency (ALT/AST > 3 times the upper limit of normal or creatinine > 221 micromol/L), severe cardiac dysfunction, thyroid dysfunction, or malignancy; (3) acute cerebrovascular disease, acute coronary syndrome, infection, or surgery within the previous 3 months; (4) left atrial thrombus confirmed by preoperative transesophageal echocardiography; (5) poor ultrasound image quality precluding speckle-tracking analysis; or (6) incomplete intraoperative left atrial voltage mapping data. The study complied with the Declaration of Helsinki and was approved by the Ethics Committee of the Second Hospital of Hebei Medical University (No. 2022-R432). Written informed consent for the ablation procedure was obtained before treatment.

**Figure 1:**
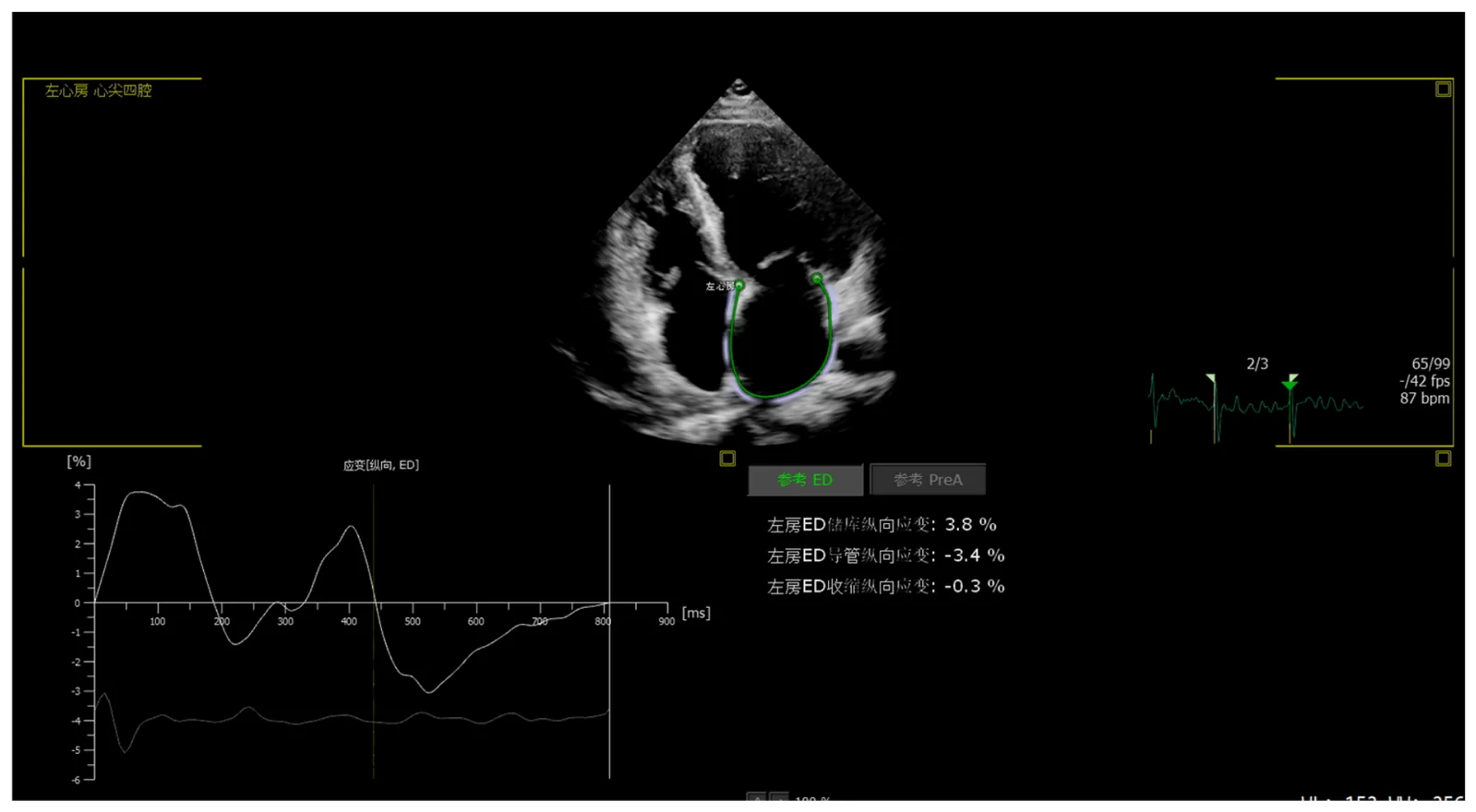
Measurement of Left Atrial Strain. The Chinese labels automatically generated by the ultrasound machine correspond to the following English terms: “Apical four-chamber view of the left atrium” (top left), “Left atrium” (green tracing), “Strain [Longitudinal, ED]” (bottom left graph), “Reference ED” and “Reference PreA” (middle buttons). The text located at the bottom right indicates “Left atrial ED reservoir longitudinal strain” (3.8%), “Left atrial ED conduit longitudinal strain” (−3.4%), and “Left atrial ED contractile longitudinal strain” (−0.3%). “ED” denotes End-Diastole.

Echocardiography and left atrial strain analysis: Echocardiography was performed using a Philips EPIQ CVx color Doppler (Philips Ultrasound, Inc., Bothell, WA, USA) ultrasound system with an S5-1 probe (1.0–5.0 MHz). Patients were examined in the left lateral decubitus position, and images were acquired during end-expiratory breath-hold. The frame rate was maintained at 60–90 frames/s. The apical four-chamber view was used to clearly display the left atrial walls and mitral annulus, with the left atrium centered in the image. Three consecutive cardiac cycles were recorded and stored. Examinations were performed before ablation and at 3 months after ablation. Conventional echocardiographic parameters included left ventricular ejection fraction (LVEF), measured using the Teichholz method; septal early diastolic mitral annular velocity (e′), measured by tissue Doppler imaging; E/e′ ratio; and LAD, measured in the parasternal long-axis view. LAS analysis was performed using AutoStrain LA software (QLAB 13.0). The region of interest was automatically generated and manually adjusted to cover the left atrial wall while excluding the pulmonary vein ostia and mitral annulus. The software tracked myocardial speckle motion and generated strain curves, with manual correction of poorly tracked segments. LAS parameters included LASr, LAScd, and LASct (Figure 1 and Table 1).

Delta values were calculated as follows:

Delta LASr = 3-month postoperative LASr − preoperative LASr;

Delta LAScd = 3-month postoperative LAScd − preoperative LAScd;

Delta LASct = 3-month postoperative LASct − preoperative LASct.

All strain measurements were performed by a single experienced sonographer who was blinded to clinical outcomes and recurrence status at the time of image analysis.

### 2.2. Radiofrequency Ablation

All procedures were performed using the CARTO three-dimensional electroanatomical mapping system and intracardiac echocardiography. Patients were placed in the supine position. After routine disinfection and sterile draping of both inguinal regions, local anesthesia was induced with 1% lidocaine, and the right femoral vein was punctured. A CARTOSOUND catheter was advanced to the right atrium to reconstruct left atrial anatomy using the CARTO system. A 10-pole coronary sinus catheter was then positioned in the distal coronary sinus, and a saline-irrigated ablation catheter (SmartTouch, Biosense Webster, Irvine, CA, USA) was advanced to the right atrium. Transseptal puncture was performed under CARTOSOUND guidance, and an 8.5-F long sheath was placed in the left atrium. Intravenous unfractionated heparin (80–100 U/kg) was administered, followed by 1000 U/h. The ablation generator was set to 40–45 W, 43 degrees C, and an irrigation flow rate of 17 mL/min.

**Figure 2:**
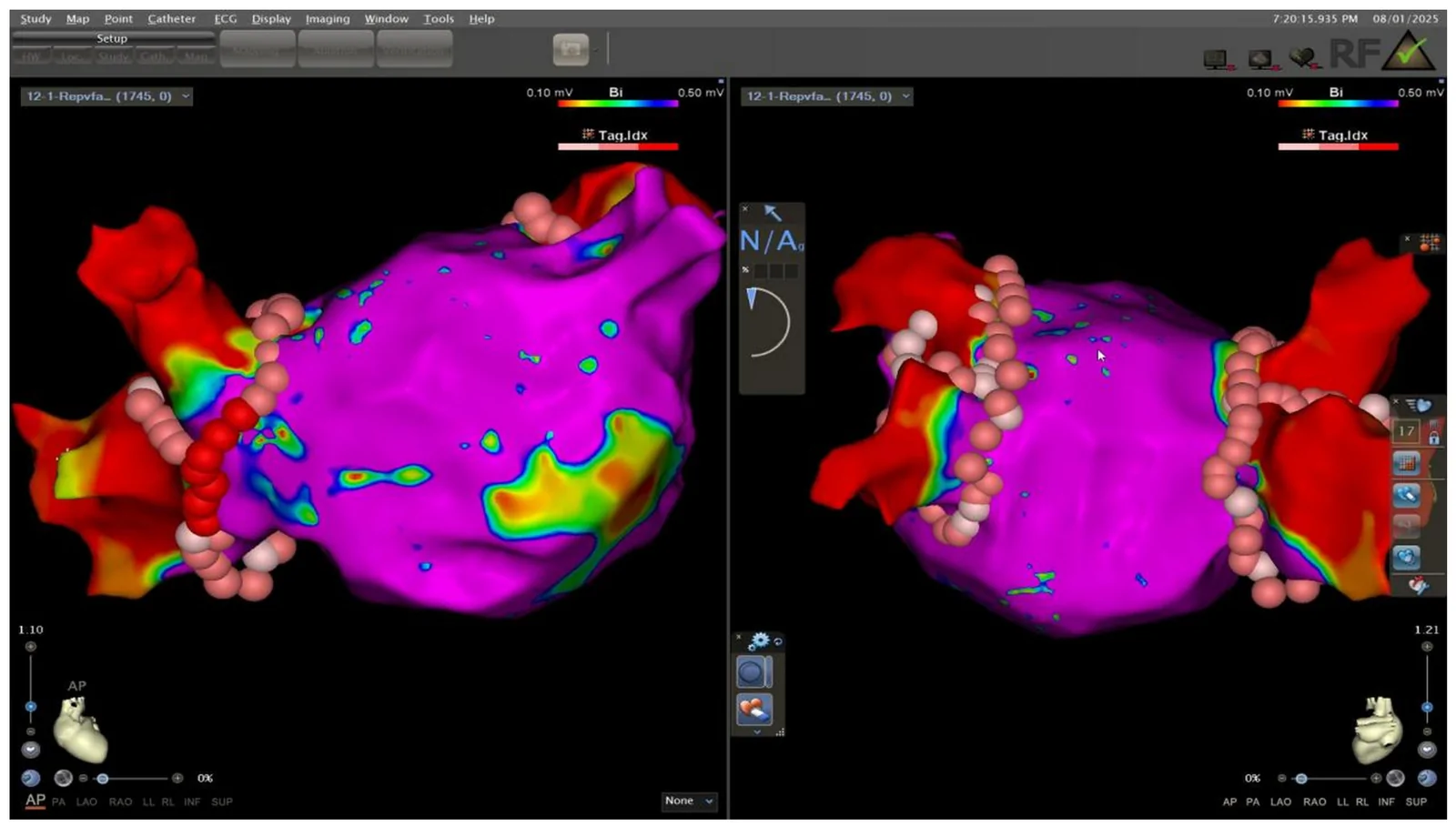
RFCA Procedure.

Circumferential pulmonary vein isolation (CPVI) was performed as the foundational ablation strategy in all patients. If sinus rhythm was not restored, ibutilide fumarate was administered or synchronized direct-current cardioversion at 200 J was performed under appropriate sedation. Left atrial voltage mapping was performed during sinus rhythm. Additional ablation, including superior vena cava isolation, cavotricuspid isthmus line, mitral isthmus line, left atrial anterior wall line, roof line, posterior wall box isolation, or substrate modification, was performed at the operator’s discretion according to the voltage mapping findings (Figure 2).

The ablation endpoint was complete electrical isolation of pulmonary vein potentials with bidirectional conduction block between the pulmonary veins and the left atrium.

### 2.3. Voltage Mapping

**Figure 3:**
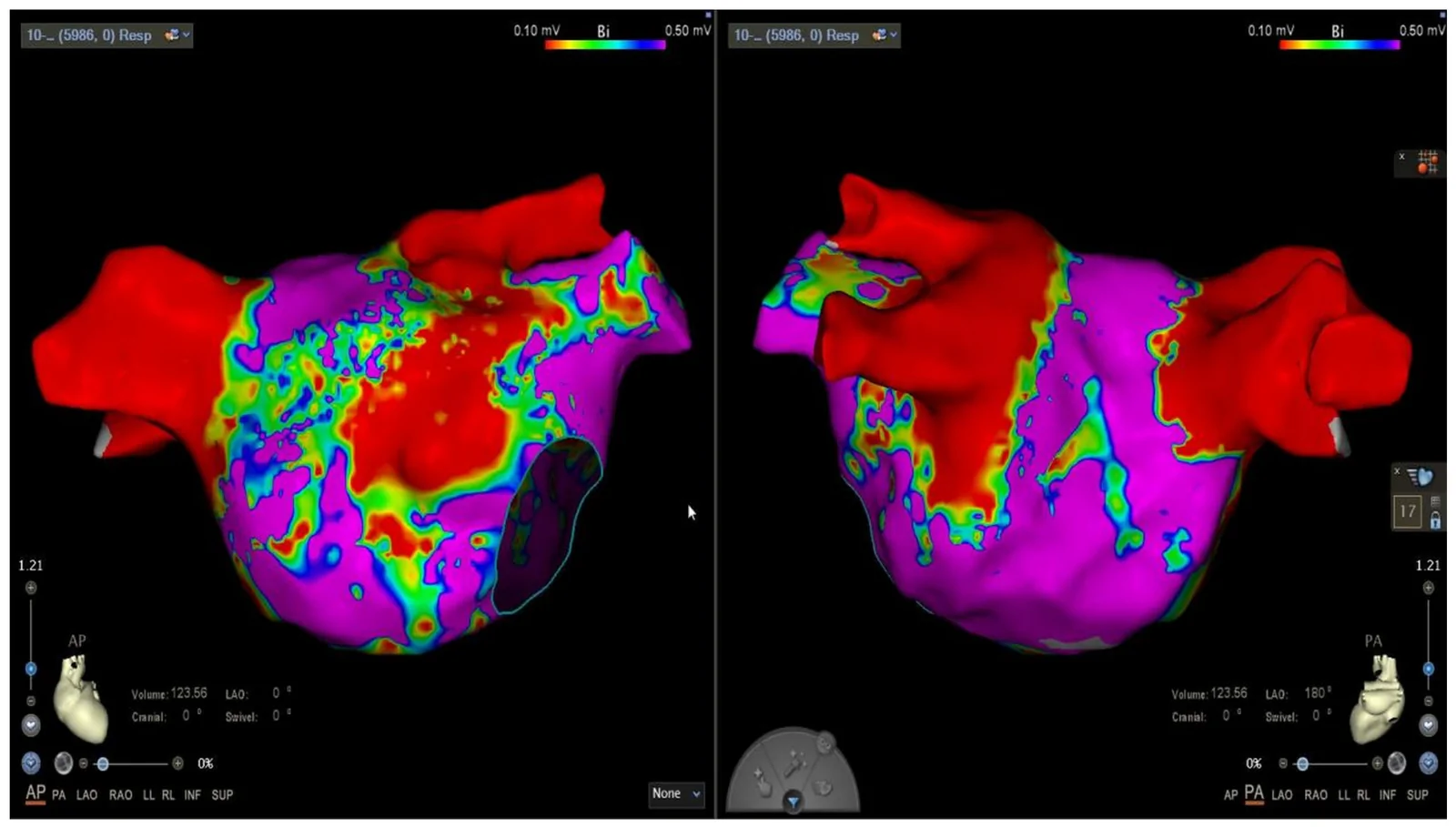
Left Atrial Voltage Map Showing Extensive Low-Voltage Zones.

**Figure 4:**
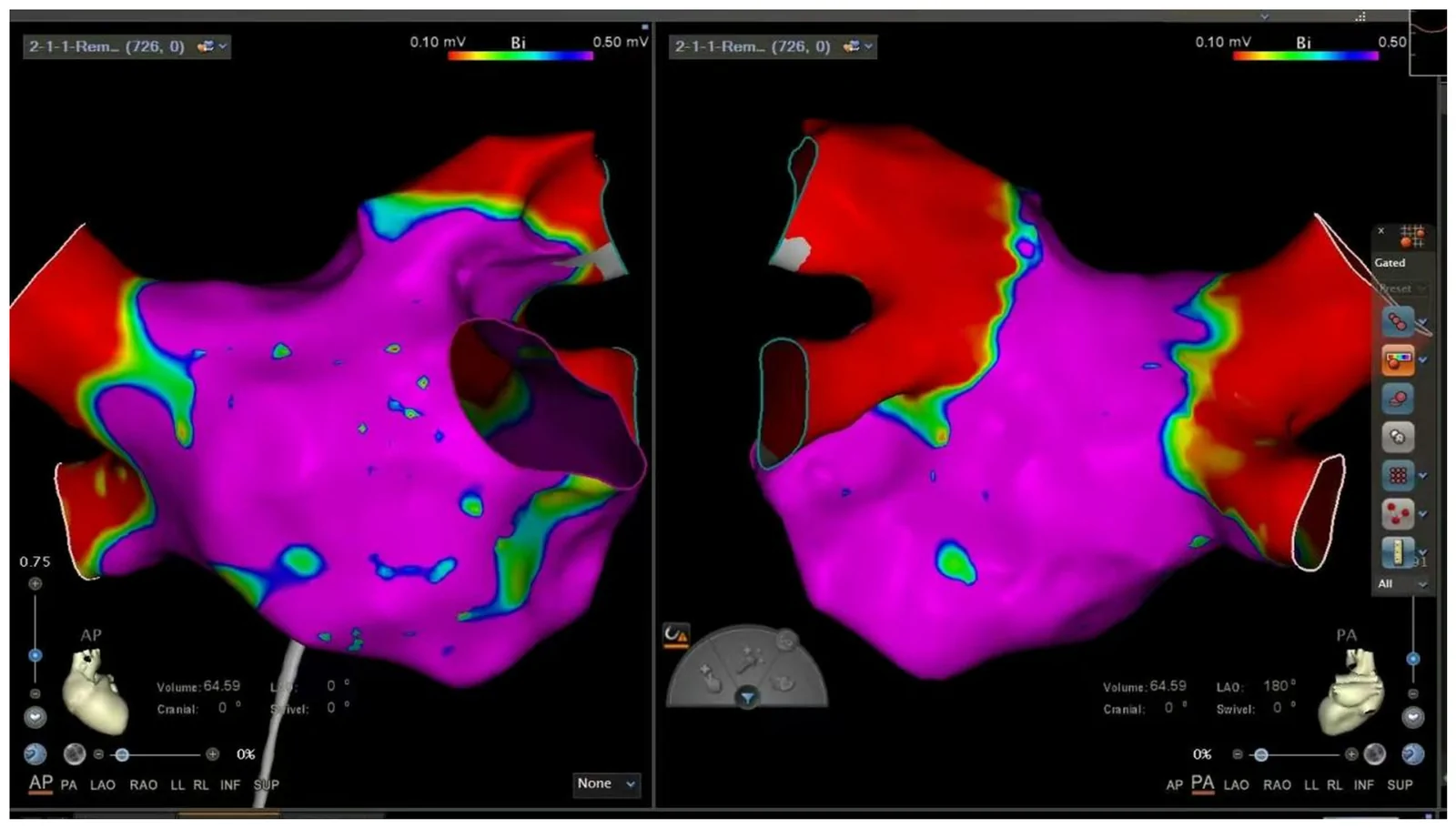
Left Atrial Voltage Map Showing Localized Low-Voltage Zones.

After restoration of sinus rhythm, left atrial voltage mapping was performed using a Pentaray high-density mapping catheter (Biosense Webster) or a Lasso catheter, with a recording-point spacing threshold of 6 mm. LVA was defined as regions with bipolar voltage amplitude < 0.5 mV during sinus rhythm [16]. Offline area-calculation software was used to calculate LVA percentage, defined as the percentage of LVA area relative to the total left atrial area (Figure 3 and Figure 4).

### 2.4. Postoperative Management and Follow-Up

Antiarrhythmic and anticoagulant therapy was continued after ablation according to clinical judgment. Follow-up was scheduled at 3 and 6 months after ablation and included assessment of clinical symptoms, 12-lead electrocardiography or 24-h Holter monitoring, and echocardiography. The 3-month follow-up visit was mandatory and included speckle-tracking imaging. The 6-month primary endpoint assessment was conducted during an outpatient visit or, in some cases, by telephone. For analyses involving LASr-3mo or Delta LASr, 3 months after ablation was defined as the landmark time. All patients included in these analyses were alive, remained under follow-up, and had an evaluable echocardiographic examination at the landmark. These analyses therefore represent dynamic postoperative risk reassessment rather than prediction from the time of ablation. Recurrence ascertainment was intermittent rather than continuous or intensive. No additional symptom-triggered ECG or Holter recordings were available in the source records for endpoint ascertainment.

The blanking period was defined as the first 3 months after ablation; arrhythmias occurring during this period were not classified as recurrence [27]. Recurrence was defined as documented atrial arrhythmia, including AF, atrial flutter, or atrial tachycardia lasting > 30 s, detected by electrocardiography or Holter monitoring after the blanking period [28]. Accordingly, the outcome for the 3-month landmark analyses was recurrence documented during the subsequent approximately 3-month interval (months 3–6 after ablation).

### 2.5. Statistical Analysis

Data were analyzed using SPSS 23.0, MedCalc version 20.0 and R language version 4.4.2. Normality was assessed using the Shapiro–Wilk test. Normally distributed continuous variables are presented as mean ± standard deviation and were compared using the independent-samples *t* test. Non-normally distributed variables are presented as median and interquartile range and were compared using the Mann–Whitney U test. Categorical variables are presented as frequencies and percentages and were compared using the chi-square test. Spearman correlation analysis was used to assess associations between LAS parameters and voltage-mapping measures. Univariate and multivariable logistic regression analyses were performed to assess factors associated with AF recurrence. Receiver operating characteristic (ROC) curve analysis was used to evaluate apparent discrimination, including the area under the curve (AUC), sensitivity, specificity, and optimal cut-off values. AUCs were compared using the DeLong test. Internal validation was performed using 1000-bootstrap resampling to estimate optimism and calculate optimism-corrected AUCs. The 95% CIs for apparent AUCs were derived via the bootstrap method (B = 1000). Calibration was assessed using bootstrap calibration curves (see Supplementary Figure S1), and optimal cut-off values were determined by the maximum Youden index. All tests were two-sided, and *p* < 0.05 was considered statistically significant.

## 3. Results

### 3.1. Baseline Characteristics

A total of 66 patients with PeAF were included. During 6 months of follow-up, 16 patients (24.2%) experienced recurrence and 50 (75.8%) remained recurrence-free. Age, sex, body mass index, AF duration, comorbidities, CHA2DS2-VASc score, and HAS-BLED score did not differ significantly between the recurrence and non-recurrence groups (all *p* > 0.05). Preoperative echocardiographic parameters, including LAD, LVEF, and E/e′ ratio, were also comparable between groups.However, LAD tended to be larger in the recurrence group than in the non-recurrence group (45.25 ± 3.86 mm vs. 42.60 ± 5.09 mm, *p* = 0.061). Preoperative LASr was significantly lower in the recurrence group than in the non-recurrence group (9.85% vs. 11.84%, *p* = 0.020), and preoperative LASct was also significantly lower in the recurrence group (−0.80% vs. −2.95%, *p* = 0.002), suggesting more severe preoperative left atrial mechanical dysfunction in patients who subsequently experienced recurrence (Table 2).

At 3 months after ablation, LASr-3mo (17.20% vs. 23.45%, *p* < 0.001) and LASct-3mo (−7.11% vs. −11.65%, *p* < 0.001) were significantly lower in the recurrence group. LAScd-3mo did not differ significantly between groups (−10.09% vs. −11.34%, *p* = 0.086). LVA percentage was significantly higher in the recurrence group (11.50% vs. 3.00%, *p* < 0.001). Delta strain analysis showed smaller improvements in the recurrence group for LASr (7.10% vs. 11.45%, *p* < 0.001), LAScd (−1.00% vs. −2.10%, *p* = 0.002), and LASct (−5.88% vs. −9.04%, *p* < 0.001) (Table 2).

### 3.2. Correlation Analysis

LASr and LASct were significantly negatively correlated at all time points (preoperative r = −0.633, postoperative r = −0.798, Delta r = −0.821; all *p* < 0.001), suggesting potential collinearity between LASr and LASct. LASr-3mo was negatively correlated with LVA (r = −0.345, *p* = 0.005), whereas LASct-3mo was positively correlated with LVA (r = 0.319, *p* = 0.009). Delta LASr was negatively correlated with LVA (r = −0.344, *p* = 0.005), and Delta LASct was positively correlated with LVA (r = 0.253, *p* = 0.040). Preoperative LASr and LASct were not significantly correlated with LVA (Table 3 and Table 4).

### 3.3. Logistic Regression Analysis

Univariate logistic regression showed that baseline clinical characteristics and conventional echocardiographic parameters were not significantly associated with recurrence. Among preoperative strain parameters, only LASct was significantly associated with recurrence (OR = 1.726, *p* = 0.010). At 3 months after ablation, LASr-3mo (OR = 0.615, *p* < 0.001) and LASct-3mo (OR = 2.599, *p* < 0.001) were both significantly associated with recurrence. Among Delta strain values, Delta LASr (OR = 0.461, *p* < 0.001), Delta LAScd (OR = 2.379, *p* = 0.007), and Delta LASct (OR = 1.699, *p* = 0.001) were all significantly associated with AF recurrence, with Delta LASr showing a strong association with recurrence among the strain-change parameters. Among voltage-mapping parameters, LVA percentage was also significantly associated with recurrence (OR = 1.081, *p* = 0.005) (Table 5).

Because LASr and LASct showed high collinearity (r = −0.798 to −0.821), LASr was selected as the representative strain parameter for multivariable modeling. This choice was based on its stronger association with recurrence in univariate analysis, the larger effect size of Delta LASr compared with Delta LASct, and the established reproducibility of LASr for global left atrial mechanical assessment. Three models were constructed: Model 1 included LASr-3mo and LVA percentage, Model 2 included Delta LASr and LVA percentage, and Model 3 included Delta LASr alone. In Model 1, LASr-3mo was an independent protective factor for AF recurrence (OR = 0.617, *p* < 0.001), whereas LVA percentage was an independent risk factor (OR = 1.069, *p* = 0.044). Each 1% increase in postoperative LASr was associated with a 38.3% reduction in the odds of recurrence. In Model 2, Delta LASr remained an independent protective factor (OR = 0.488, *p* = 0.001), whereas LVA was no longer statistically significant (OR = 1.034, *p* = 0.307). Model 3, which included Delta LASr alone, had a predictive accuracy of 83.3% (Table 6).

### 3.4. ROC Curve Analysis and Predictive Performance

**Figure 5:**
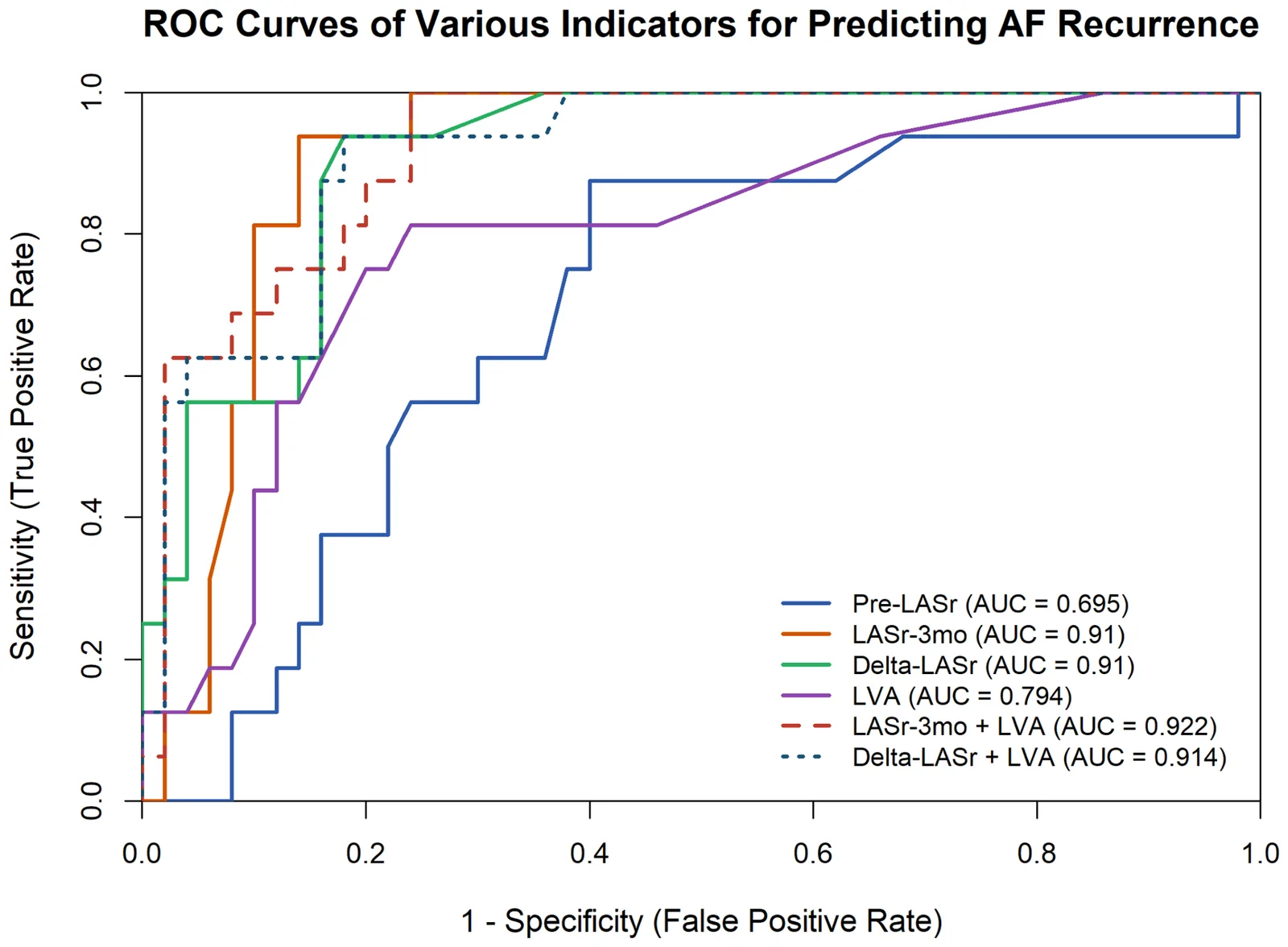
ROC Curves of Indicators for Predicting Atrial Fibrillation Recurrence.

ROC analysis showed limited predictive performance for preoperative LASr (Apparent AUC = 0.695). Predictive performance improved substantially for LASr-3mo (Apparent AUC = 0.910; sensitivity, 93.8%; specificity, 86.0%; optimal cut-off, 19.95%). Delta LASr also showed excellent predictive performance (Apparent AUC = 0.913; sensitivity, 93.8%; specificity, 84.0%; optimal cut-off, 9.6%) and was comparable to LASr-3mo (*p* = 0.936). LVA alone showed moderate predictive performance (Apparent AUC = 0.794). The combined LASr-3mo + LVA model had the highest apparent AUC (0.923) and achieved 100% sensitivity with 76.0% specificity. The apparent AUC of Delta LASr + LVA was 0.914.(Table 7 and Figure 5).

Bootstrap internal validation indicated modest optimism: corrected AUCs were 0.913 for Model 1 (LASr-3mo + LVA) and 0.906 for Model 2 (Delta LASr + LVA), with optimism of 0.010 and 0.008, respectively. Calibration slopes were 0.88 and 0.91, suggesting good calibration (Table 7). Sensitivity and specificity with 95% confidence intervals are provided in Table 7.

DeLong testing showed no significant difference between LASr-3mo and Delta LASr (*p* = 0.936). Delta LASr was superior to LVA alone (*p* = 0.047). The LASr-3mo + LVA model had a numerically higher AUC than LASr-3mo alone, but the difference was not statistically significant (*p* = 0.602). Delta LASr + LVA did not differ significantly from Delta LASr alone (*p* = 0.922). The two combined models showed comparable performance (*p* = 0.805) (Table 8).

### 3.5. LVA Stratification Analysis

Using the optimal LVA cut-off of 4.5%, patients were divided into a low-LVA group (≤4.5%, *n* = 41) and a high-LVA group (>4.5%, *n* = 25). The high-LVA group was older (69.44 ± 6.37 vs. 60.29 ± 10.07 years, *p* < 0.001), had higher CHA2DS2-VASc (*p* = 0.019) and HAS-BLED scores (*p* = 0.001), had a higher frequency of previous stroke (36.0% vs. 9.8%, *p* = 0.023), and larger preoperative LAD (45.44 ± 3.79 vs. 41.90 ± 5.10 mm, *p* = 0.004). Postoperative LASr-3mo (19.39% vs. 23.49%, *p* = 0.001) and LASct-3mo (−8.84% vs. −11.60%, *p* = 0.001) were lower in the high-LVA group, indicating poorer functional recovery. The high-LVA group showed less improvement in Delta LASr than the low-LVA group (8.60% vs. 11.80%, *p* = 0.001), and Delta LASct was also significantly lower (−6.98% vs. −9.09%, *p* = 0.002). Regarding recurrence, the 6-month recurrence rate was significantly higher in the high-LVA group than in the low-LVA group (52.0% vs. 7.3%, *p* < 0.001) (Table 9).

**Figure 6:**
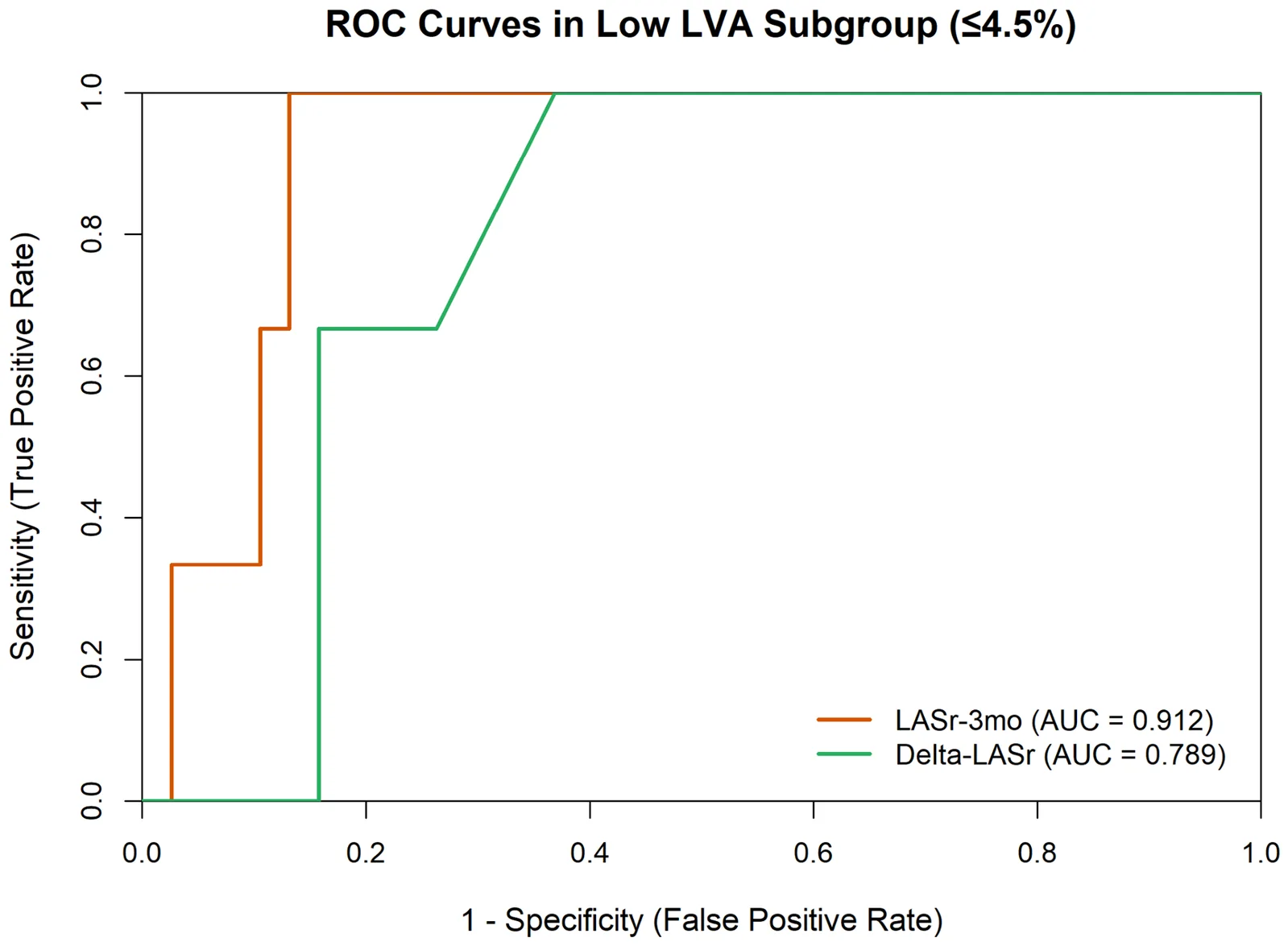
ROC Curves of Delta LASr and LASr-3mo in the Low-LVA Subgroup (LVA ≤ 4.5%, *n* = 41; recurrence, *n* = 3).

**Figure 7:**
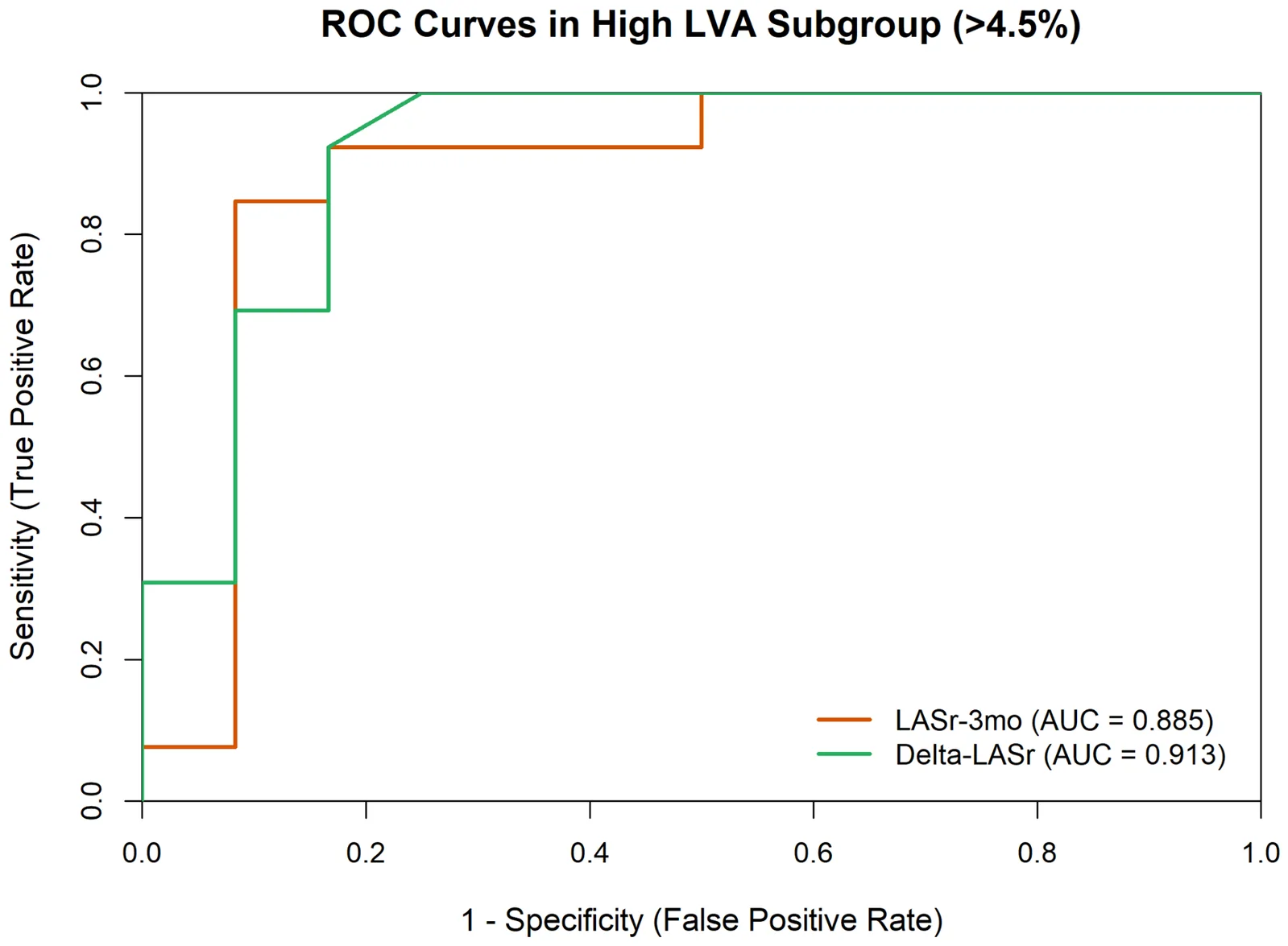
ROC Curves of Delta LASr and LASr-3mo in the High-LVA Subgroup (LVA > 4.5%, *n* = 25; recurrence, *n* = 13).

To further evaluate the predictive performance of Delta LASr and LASr-3mo across LVA subgroups, DeLong testing compared the high-LVA subgroup (*n* = 25, with 13 recurrences) with the entire cohort. The low-LVA subgroup had only 3 recurrence events, making ROC estimates unstable. In the high-LVA group, Delta LASr predicted recurrence with an AUC of 0.917 (sensitivity, 100%; specificity, 83.3%), comparable to the entire cohort (AUC = 0.913; DeLong *p* = 0.962). LASr-3mo had an AUC of 0.885 in the high-LVA group and showed similar discriminatory performance to that in the entire cohort (DeLong *p* > 0.05; Table 10 and Figure 6 and Figure 7), although these subgroup ROC estimates should be interpreted cautiously because of the small number of recurrence events.

## 4. Discussion

Patients with PeAF generally have more advanced structural and electrical remodeling than those with paroxysmal AF, and recurrence after ablation remains common [29]. Whereas paroxysmal AF is often driven predominantly by pulmonary vein triggers, PeAF is more strongly associated with left atrial substrate abnormalities, including fibrosis, conduction heterogeneity, and impaired mechanical function [5]. In this population, we evaluated the relationships among LAS, LVA, and recurrence after RFCA and explored combined electro-mechanical models.

Preoperative LASr and LASct were reduced in the recurrence group, whereas LAScd did not differ significantly. These findings suggest that active left atrial mechanical function is impaired before ablation in patients who later experience recurrence, while passive conduit function may be relatively preserved. This pattern is consistent with the concept that left ventricular longitudinal systolic function strongly influences LASr and that left atrial contractile function directly affects LASct [30]. In PeAF, long-standing rapid and irregular atrial activation can promote myocardial energy depletion, abnormal calcium handling, and contractile-protein remodeling, which may first affect active atrial contraction and relaxation [31]. However, preoperative LASr showed only limited predictive value in this cohort (AUC = 0.695), suggesting that dynamic postoperative recovery may be more informative than baseline function alone in PeAF.

We selected 3 months after ablation as the landmark assessment time point because it corresponds to the end of the blanking period and the early phase of left atrial reverse remodeling [32]. LASr improved substantially in the non-recurrence group (from 11.84% to 23.45%), whereas improvement was limited in the recurrence group (from 9.85% to 17.20%). These findings are consistent with observations in long-standing persistent AF and support postoperative left atrial functional recovery as an early marker of ablation success [33]. Importantly, LASr-3mo incorporates information about the early response to ablation and reverse remodeling and should not be interpreted in the same manner as a pretreatment risk marker.

LVA was significantly and negatively correlated with LASr-3mo and remained independently associated with recurrence in the LASr-3mo + LVA model. These findings support the concept that electrical substrate abnormalities constrain mechanical recovery: fibrotic low-voltage regions have reduced electrical activity and impaired local contractile function, thereby compromising global left atrial performance [34]. Previous work comparing strain imaging with high-density voltage mapping showed spatial correspondence between reduced strain and low-voltage regions [35]. In our cohort, patients with LVA > 4.5% showed less postoperative LASr recovery and a higher observed recurrence proportion; however, the cohort-derived threshold and subgroup estimates remain exploratory.

These substrate-based findings should also be interpreted within the broader heterogeneity of AF phenotypes. AF is not a uniform clinical entity, and its mode of presentation, timing of diagnosis, comorbidity profile, and clinical trajectory may identify populations with distinct substrate characteristics and prognoses. Recent prospective registry evidence comparing first-diagnosed with previously known AF demonstrated clinically relevant differences in patient characteristics and outcomes [36]. Although that study did not evaluate recurrence after ablation and should not be regarded as direct evidence for the present endpoint, it provides important context for our findings. LAS and LVA should therefore not be viewed as isolated determinants of post-ablation risk, but as potential components of a multidimensional assessment that also incorporates AF phenotype, disease history, comorbidity burden, and longitudinal clinical course.

Delta LASr showed better apparent discrimination than preoperative LASr in this cohort (AUC = 0.913 vs. 0.695). This dynamic marker integrates baseline functional status with postoperative recovery and may therefore reflect the early response of left atrial function to ablation. Because Delta LASr incorporates information obtained during the first 3 months after ablation, its association with recurrence represents risk reassessment at the 3-month landmark rather than baseline prediction. The cohort-derived cut-off of 9.6% is exploratory and should not be used in isolation to determine follow-up or treatment.

LASr and LASct were highly correlated at each time point, raising a potential collinearity issue for multivariable modeling. Rather than including all strain parameters simultaneously, we selected LASr for model construction because it showed strong associations with recurrence, reflects global left atrial mechanical function, and has favorable reproducibility [37]. This approach improved model interpretability while limiting the simultaneous inclusion of correlated strain measures.

The combined LASr-3mo + LVA model showed the highest apparent discrimination in this cohort (AUC = 0.923; sensitivity, 100%; specificity, 76.0%). The Delta LASr + LVA model also showed high apparent discrimination (AUC = 0.914; sensitivity, 93.8%; specificity, 82.0%), and the two combined models were comparable. Although adding LVA did not significantly increase the AUC relative to LASr-3mo alone, it increased apparent sensitivity in this dataset. These results suggest that postoperative mechanical function and electrical substrate may provide complementary information, but the models remain exploratory because performance was estimated in the same small cohort in which they were developed.

From a clinical perspective, these findings support further evaluation of integrated electro-mechanical assessment rather than the immediate adoption of isolated LAS or LVA cut-offs. The LASr-3mo and LVA thresholds identified in this cohort should not independently determine rhythm-monitoring intensity, pharmacological management, or repeat-ablation strategy. If externally validated, LAS and LVA may complement established clinical factors and the broader AF phenotype in multidimensional risk assessment.

The optimal LVA cut-off for recurrence in this cohort was 4.5%, lower than the 10% threshold commonly used in previous studies. This difference may reflect the small sample size, the distribution of LVA burden in this PeAF cohort, and variation in mapping methodology. Because the 4.5% value was derived and evaluated in the same cohort, it should be regarded as exploratory and cohort-specific rather than as a clinically actionable decision threshold. External validation is required before either this value or other LVA cut-offs can be incorporated into post-ablation management.

After stratification by the 4.5% cut-off, recurrence was substantially more common in the high-LVA group than in the low-LVA group (52.0% vs. 7.3%, *p* < 0.001). Patients in the high-LVA group were older, had higher CHA2DS2-VASc and HAS-BLED scores, a higher prevalence of previous stroke, larger preoperative LAD, and poorer recovery of LASr and LASct. These findings are consistent with reports that LVA predicts recurrence after AF ablation and with meta-analytic evidence showing that LVA is associated with increased recurrence risk [38,39]. Together with biopsy and mapping studies linking LVA to atrial fibrosis [40], our findings are compatible with an interplay among structural substrate, mechanical function, and clinical outcome, although causal direction cannot be established from this study.

This study has several strengths. It focused specifically on patients with PeAF, a population with more advanced substrate remodeling and higher recurrence risk than mixed or paroxysmal AF cohorts [41,42]. The 3-month postoperative assessment captured functional status at the end of the blanking period and enabled landmark-based evaluation of subsequent recurrence. By combining 2D-STE-derived mechanical measures with EAM-derived electrical substrate assessment, the study explored a complementary electro-mechanical framework for postoperative risk reassessment. The subgroup findings further suggest that functional measures may remain informative across different levels of LVA burden, although these estimates are preliminary.

This study also has limitations. Its single-center, retrospective design and modest sample size (*n* = 66) introduce the possibility of selection bias and limit generalizability. Follow-up was limited to 6 months, which may have resulted in underestimation of later recurrence. Atrial fibrosis was not independently assessed using late gadolinium-enhanced cardiac magnetic resonance, and other potentially informative measures, including left atrial stiffness, atrial mechanical dispersion, and artificial intelligence-assisted echocardiographic parameters, were not evaluated [43]. The high-LVA subgroup was small (*n* = 25, including 13 patients with recurrence), and only three recurrence events occurred in the low-LVA subgroup, limiting the precision and stability of the ROC estimates. The findings therefore require validation in larger, multicenter prospective studies with longer follow-up. LASr-3mo and Delta LASr must also be interpreted within the landmark design: they represent risk reassessment at 3 months for recurrence during the subsequent approximately 3 months, rather than prediction from the time of ablation. Recurrence ascertainment relied on scheduled 12-lead ECG or 24-h Holter monitoring at 3 and 6 months, with some 6-month assessments conducted by telephone. Asymptomatic or intermittent arrhythmias between scheduled assessments may therefore have been missed. Because no additional symptom-triggered ECG or Holter recordings were available in the source records, the observed recurrence proportion of 24.2% reflects recurrence detected under the study protocol and should not be equated with the overall AF recurrence rate or AF burden that might have been identified through continuous or more intensive rhythm monitoring.

Interpretation of the association between LVA and recurrence is further complicated by the heterogeneous, non-randomized ablation strategy. Although all patients underwent CPVI, additional lesion sets and substrate-modification procedures were selected at the operator’s discretion according to voltage-mapping findings. LVA may consequently have influenced both treatment selection and subsequent recurrence risk, creating a potential treatment-by-substrate interaction. The observed association should therefore not be interpreted as a purely prognostic or causal relationship and may partly reflect confounding by treatment selection. The frequencies of individual additional ablation procedures could not be reliably reconstructed from the available procedural records, and the small number of recurrence events precluded a stable treatment-adjusted sensitivity analysis. LVA should thus be regarded as an exploratory observational marker requiring confirmation in larger prospective cohorts with prespecified, standardized ablation protocols and systematic documentation of additional lesion sets.

Voltage mapping was performed using either a Pentaray or a Lasso catheter. Differences in electrode configuration and sampling density between these technologies may have introduced heterogeneity into LVA estimation. Because patient-level catheter allocation could not be reliably reconstructed from the available database, catheter-specific patient numbers and comparisons of LVA percentage by mapping technology could not be reported. Equivalence between the two mapping approaches should therefore not be inferred.

Intraobserver and interobserver reproducibility of strain measurements was not assessed. This is particularly relevant because relatively small differences in postoperative strain contributed to the predictive models, and measurement variability may have affected the stability of the estimates. The strain-based findings and model performance should therefore be regarded as exploratory pending replication in studies incorporating prespecified repeat measurements by the same reader and by independent readers.

## 5. Conclusions

At the 3-month landmark after RFCA, LASr-3mo was associated with recurrence during the subsequent approximately 3-month interval in patients with PeAF. Because LASr-3mo and Delta LASr incorporate the early response to ablation and reverse remodeling, they should be interpreted as dynamic postoperative risk-reassessment markers rather than pretreatment predictors. LVA showed an exploratory association with recurrence and with impaired postoperative recovery of left atrial function. The combined LASr-3mo + LVA model showed promising apparent discrimination, but its performance was estimated in the same small cohort and requires internal and external validation. LVA and the cohort-derived cut-offs should be regarded as exploratory observational findings and should not currently direct surveillance or treatment. Any future clinical application should integrate these measures with the broader AF phenotype, comorbidity profile, and longitudinal clinical course. The observed recurrence proportion reflects protocol-detected recurrence rather than total AF burden.

## Figures and Tables

**Table 1:** Definitions and Calculation of Left Atrial Strain Parameters.

Parameter	Definition	Calculation Formula
Left atrial reservoir strain (LASr)	Longitudinal elongation rate of the left atrium during left ventricular systole	(Maximum LA length − Minimum LA length)/Minimum LA length × 100%, expressed as positive value
Left atrial conduit strain (LAScd)	Longitudinal shortening rate of the left atrium during early left ventricular diastole	(Maximum LA length − Early diastolic LA length)/Maximum LA length × 100%, expressed as negative value
Left atrial contraction strain (LASct)	Active shortening rate of the left atrium during left atrial contraction	(Early diastolic LA length − End-systolic LA length)/Early diastolic LA length × 100%, expressed as negative value

**Table 2:** Baseline Characteristics of the Recurrence and Non-Recurrence Groups.

	Non-Recurrence Group (*n* = 50)	Recurrence Group (*n* = 16)	*p* Value
Age (years)	63.38 ± 10.46	64.94 ± 7.94	0.587
Male, *n* (%)	33 (66.0)	11 (68.8)	0.839
BMI (kg/m^2^)	26.37 ± 3.66	26.70 ± 3.36	0.746
AF duration (months)	18.00 (1.00, 59.00)	6.00 (1.00, 60.00)	0.517
Coronary heart disease, *n* (%)	7 (14.0)	2 (12.5)	0.879
Hypertension, *n* (%)	26 (52.0)	11 (68.8)	0.240
Diabetes mellitus, *n* (%)	10 (20.0)	3 (18.8)	0.913
Stroke, *n* (%)	8 (16.0)	5 (31.3)	0.182
CHA2DS2-VASc score	3.00 (1.00, 5.00)	3.00 (2.00, 4.00)	0.862
HAS-BLED score	1.00 (0.00, 2.00)	2.00 (1.00, 3.00)	0.109
LAD (mm)	42.60 ± 5.09	45.25 ± 3.86	0.061
LVEF (%)	62.40 (58.99, 63.32)	62.55 (56.42, 65.35)	0.958
E/e′ ratio	12.00 (9.80, 15.50)	12.20 (10.13, 18.55)	0.875
Preoperative LASr (%)	11.84 (9.93, 13.78)	9.85 (8.35, 11.65)	0.020 *
Preoperative LAScd (%)	−9.40 (−11.23, −8.40)	−9.00 (−10.53, −8.00)	0.765
Preoperative LASct (%)	−2.95 (−4.90, −2.00)	−0.80 (−1.45, −0.80)	0.002 *
LASr-3mo (%)	23.45 ± 4.45	17.20 ± 2.15	<0.001 *
LAScd-3mo (%)	−11.34 ± 2.68	−10.09 ± 1.78	0.086
LASct-3mo (%)	−11.65 ± 2.45	−7.11 ± 1.58	<0.001 *
LVA percentage (%)	3.00 (2.00, 4.25)	11.50 (5.50, 24.25)	<0.001 *
Delta LASr (%)	11.45 (9.35, 13.27)	7.10 (5.85, 9.10)	<0.001 *
Delta LAScd (%)	−2.10 (−3.02, −1.10)	−1.00 (−1.65, −1.00)	0.002 *
Delta LASct (%)	−9.04 ± 2.59	−5.88 ± 1.91	<0.001 *

Note: * *p* < 0.05. LAD, left atrial diameter; LASr, left atrial reservoir strain; LAScd, left atrial conduit strain; LASct, left atrial contraction strain; 3mo, 3 months postoperatively; Delta, difference between 3-month postoperative and preoperative values; LVA, low-voltage area.

**Table 3:** Correlations Among Left Atrial Strain Parameters.

Period	Variable	LASr	LASct	LAScd
Preoperative	LASr	1.000	−0.633	—
	LASct	−0.633	1.000	—
3 months postoperative	LASr	1.000	−0.798	—
	LASct	−0.798	1.000	—
Delta	LASr	1.000	−0.821	−0.475
	LASct	−0.821	1.000	0.096
	LAScd	−0.475	0.096	1.000

Note: A dash indicates that the correlation was not analyzed for the corresponding period. LAScd was significantly correlated with LVA only for Delta values (*p* = 0.038), with no significant correlation before ablation or at 3 months after ablation; therefore, only Delta LAScd was included in the correlation analysis.

**Table 4:** Correlations Between Left Atrial Strain Parameters and LVA.

Parameter	Correlation Coefficient	*p* Value
Preoperative LASr-%	−0.109	0.384
Preoperative LASct-%	0.125	0.316
LASr-3mo%	−0.345	0.005 *
LASct-3mo%	0.319	0.009 *
Delta LASr	−0.344	0.005 *
Delta LAScd	0.162	0.195
Delta LASct	0.253	0.040 *

Note: * *p* < 0.05 was considered statistically significant.

**Table 5:** Univariate Logistic Regression Analysis of Factors Associated With Atrial Fibrillation Recurrence.

Variable	B	SE	*p* Value	OR	95% CI
Age	0.017	0.03	0.581	1.017	0.958–1.079
Sex	0.125	0.616	0.839	1.133	0.339–3.794
BMI	0.027	0.081	0.742	1.027	0.876–1.205
AF duration	−0.005	0.006	0.462	0.995	0.983–1.008
Coronary heart disease	−0.131	0.859	0.879	0.878	0.163–4.724
Hypertension	0.708	0.609	0.245	2.031	0.615–6.701
Diabetes mellitus	−0.08	0.732	0.913	0.923	0.220–3.872
Stroke	0.87	0.663	0.190	2.386	0.651–8.753
LAD	0.119	0.065	0.066	1.127	0.992–1.279
LVEF	−0.005	0.037	0.900	0.995	0.926–1.070
E/e′	−0.002	0.037	0.953	0.998	0.928–1.072
LASr	−0.154	0.092	0.095	0.857	0.716–1.027
LAScd	0.025	0.111	0.820	1.025	0.826–1.273
LASct	0.566	0.221	0.010 *	1.726	1.143–2.717
LASr-3mo	−0.486	0.130	<0.001 *	0.615	0.477–0.794
LAScd-3mo	0.207	0.122	0.090	1.229	0.968–1.562
LASct-3mo	0.955	0.254	<0.001 *	2.599	1.580–4.277
Delta LASr	−0.775	0.212	<0.001 *	0.461	0.304–0.698
Delta LAScd	0.867	0.322	0.007 *	2.379	1.266–4.469
Delta LASct	0.530	0.153	0.001 *	1.699	1.259–2.292
LVA	0.077	0.028	0.005 *	1.081	1.024–1.141

Note: * *p* < 0.05 was considered statistically significant.

**Table 6:** Multivariable Logistic Regression Analysis of Risk Factors for Atrial Fibrillation Recurrence.

Variable	B	SE	*p* Value	OR	95% CI
LASr-3mo	−0.482	0.137	<0.001	0.617	0.472–0.807
LVA	0.066	0.033	0.044	1.069	1.002–1.140
Delta LASr	−0.718	0.213	0.001	0.488	0.321–0.740
LVA	0.034	0.033	0.307	1.034	0.969–1.104
Delta LASr	−0.775	0.212	<0.001	0.461	0.304–0.698

Note: Model 1 predictive accuracy 84.8%, Model 2 predictive accuracy 86.4%, Model 3 predictive accuracy 83.3%.

**Table 7:** ROC Curve Analysis of Indicators for Predicting Atrial Fibrillation Recurrence.

Predictor	Apparent AUC	Corrected AUC (95% CI)	Calibration Slope	Sensitivity (95% CI), %	Specificity (95% CI), %	Optimal Cut-Off Value
LASr	0.695	0.697 (0.552–0.826)	2.344	87.5 (61.7–98.4)	60.0 (45.2–73.6)	11.85
LASr-3mo	0.910	0.910 (0.832–0.974)	0.996	93.8 (69.8–99.8)	86.0 (73.3–94.2)	19.95
Delta LASr	0.913	0.911 (0.841–0.968)	0.98	87.5 (61.7–98.4)	84.0 (70.9–92.8)	9.6
LVA	0.794	0.793 (0.657–0.905)	1.075	81.3 (54.4–96.0)	76.0 (61.8–86.9)	4.5
LASr-3mo + LVA	0.923	0.913 (0.703–0.973)	0.88	100.0 (79.4–100.0)	76.0 (61.8–86.9)	0.131
Delta LASr + LVA	0.914	0.906 (0.677–0.970)	0.91	93.8 (69.8–99.8)	82.0 (68.6–91.4)	0.206

Note: Apparent AUCs and 95% CIs were calculated using the bootstrap method (B = 1000). Corrected AUCs were derived from 1000-bootstrap resampling. Calibration slopes were obtained from bootstrap calibration curves. Sensitivity and specificity are reported at the optimal cut-off (maximum Youden index) with 95% CIs. For combined models, the cut-off value refers to the predicted probability from logistic regression.

**Table 8:** Comparison of Predictive Performance Between Combined Models and Individual Indicators.

Comparison	Apparent AUC_1_	Apparent AUC_2_	Z Value	*p* Value
LASr-3mo vs Delta LASr	0.910	0.913	−0.080	0.936
LASr-3mo vs LVA	0.910	0.794	1.524	0.128
Delta LASr vs LVA	0.913	0.794	1.950	0.047 *
LASr-3mo + LVA vs LASr-3mo	0.923	0.910	0.521	0.602
Delta LASr + LVA vs Delta LASr	0.914	0.913	0.098	0.922
LASr-3mo + LVA vs Delta LASr + LVA	0.923	0.914	0.248	0.805

Note: * *p* < 0.05 was considered statistically significant.

**Table 9:** Baseline Characteristics of the Low- and High-LVA Groups.

Parameter	Low LVA Group	High LVA Group	*p* Value
	(≤4.5%, *n* = 41)	(>4.5%, *n* = 25)	
Age (years)	60.29 ± 10.07	69.44 ± 6.37	<0.001 *
Male, *n* (%)	30 (73.2)	14 (56)	0.184
BMI (kg/m^2^)	26.99 ± 3.38	25.55 ± 3.75	0.113
AF duration (months)	7.00 (1.00, 60.00)	24.00 (2.00, 60.00)	0.389
CHA_2_DS_2_-VASc score	2.00 (1.00, 4.00)	4.00 (2.50, 4.50)	0.019 *
HAS-BLED score	1.00 (0.00, 2.00)	2.00 (1.00, 2.50)	0.001 *
Coronary heart disease	6 (14.6)	3 (12)	1
Hypertension	20 (48.8)	17 (68)	0.201
Diabetes mellitus	9 (22)	4 (16)	0.752
Stroke	4 (9.8)	9 (36)	0.023 *
Preoperative LAD (mm)	41.90 ± 5.10	45.44 ± 3.79	0.004 *
Preoperative LVEF (%)	61.60 (59.80, 65.70)	62.80 (58.90, 64.75)	0.974
E/e′ ratio	11.50 (9.60, 15.57)	13.10 (10.12, 16.60)	0.341
Preoperative LASr	11.70 ± 3.20	10.78 ± 3.19	0.261
Preoperative LAScd	−9.40 (−10.35, −7.95)	−9.30 (−10.45, −7.70)	0.900
Preoperative LASct	−2.90 (−3.85, −1.00)	−1.00 (−3.60, −0.70)	0.075
LASr-3mo	23.49 ± 4.78	19.39 ± 3.76	0.001 *
LAScd-3mo	−11.33 ± 2.68	−10.56 ± 2.25	0.234
LASct-3mo	−11.60 ± 2.51	−8.84 ± 2.97	0.001 *
Delta LASr	11.80 (10.00, 14.05)	8.60 (6.35, 10.75)	0.001 *
Delta LAScd	−2.14 ± 1.26	−1.61 ± 1.32	0.113
Delta LASct	−9.09 ± 2.58	−6.98 ± 2.68	0.002 *
Recurrence, *n* (%)	3 (7.3)	13 (52.0)	<0.001 *

Note: * *p* < 0.05 was considered statistically significant.

**Table 10:** Predictive Performance of Delta LASr and LASr-3mo Across LVA Subgroups.

Predictor	Subgroup	AUC	Sensitivity	Specificity	Optimal Cut-Off Value	DeLong Test
Delta LASr	Low LVA group	0.807	100%	65.8%	10.10%	
	High LVA group	0.917	100%	83.3%	9.6%	*p* = 0.962
	Entire cohort	0.913	93.8%	84.0%	9.6%	
LASr-3mo	Low LVA group	0.912	100%	86.8%	20.15%	
	High LVA group	0.885	84.6%	91.7%	18.75%	*p* = 0.779
	Entire cohort	0.910	93.8%	86.0%	19.95%	

Note: The low-LVA group had only 3 recurrence events; therefore, ROC estimates in this subgroup should be interpreted cautiously. DeLong tests compared the high-LVA subgroup with the entire cohort.

## Data Availability

The data presented in this study are available on request from the corresponding author due to privacy and ethical restrictions.

## Supplementary Materials

The following supporting information can be downloaded at: https://www.mdpi.com/article/10.3390/diagnostics16203303/s1, Figure S1: Calibration curve for Model 1. The blue dashed line represents the ideal calibration (45-degree reference line). The red solid line represents the apparent calibration. The green solid line represents the bias-corrected calibration via 1000 Bootstrap resamples; Figure S2: Calibration curve for Model 2. The blue dashed line represents the ideal calibration (45-degree reference line). The red solid line represents the apparent calibration. The green solid line represents the bias-corrected calibration via 1000 Bootstrap resamples.

## Abbreviations

**Abbreviation**	**Full Name**
AF	Atrial fibrillation
RFCA	Radiofrequency catheter ablation
PeAF	Persistent atrial fibrillation
PAF	Paroxysmal AF
EAM	Electroanatomical mapping
LVA	Low-voltage area
LAD	Left atrial diameter
LAVI	Left atrial volume index
2D-STE	Two-dimensional speckle-tracking echocardiography
LAS	Left atrial strain
LASr	Left atrial reservoir strain
LAScd	Left atrial conduit strain
LASct	Left atrial contraction strain
CPVI	Circumferential pulmonary vein isolation
BMI	Body Mass Index
LVEF	Left ventricular ejection fraction
CMR	Cardiac magnetic resonance

## References

[1] National Center for Cardiovascular Diseases (2025). Summary of China cardiovascular health and disease report 2024. Chin. Circ. J..

[2] Becher N., Metzner A., Toennis T., Kirchhof P., Schnabel R.B. (2024). Atrial fibrillation burden: A new outcome predictor and therapeutic target. Eur. Heart J..

[3] Roth G.A., Mensah G.A., Johnson C.O., Addolorato G., Ammirati E., Baddour L.M., Barengo N.C., Beaton A.Z., Benjamin E.J., Benziger C.P. (2020). Global Burden of Cardiovascular Diseases and Risk Factors, 1990–2019: Update From the GBD 2019 Study. J. Am. Coll. Cardiol..

[4] Schnabel R.B., Marinelli E.A., Arbelo E., Boriani G., Boveda S., Buckley C.M., Camm A.J., Casadei B., Chua W., Dagres N. (2023). Early diagnosis and better rhythm management to improve outcomes in patients with atrial fibrillation: The 8th AFNET/EHRA consensus conference. Europace.

[5] Calkins H., Hindricks G., Cappato R., Kim Y.H., Saad E.B., Aguinaga L., Akar J.G., Badhwar V., Brugada J., Camm J. (2017). 2017 HRS/EHRA/ECAS/APHRS/SOLAECE expert consensus statement on catheter and surgical ablation of atrial fibrillation. Heart Rhythm..

[6] January C.T., Wann L.S., Calkins H., Chen L.Y., Cigarroa J.E., Cleveland J.C., Ellinor P.T., Ezekowitz M.D., Field M.E., Furie K.L. (2019). 2019 AHA/ACC/HRS Focused Update of the 2014 AHA/ACC/HRS Guideline for the Management of Patients with Atrial Fibrillation: A Report of the American College of Cardiology/American Heart Association Task Force on Clinical Practice Guidelines and the Heart Rhythm Society in Collaboration with the Society of Thoracic Surgeons. Circulation.

[7] Joglar J.A., Chung M.K., Armbruster A.L., Benjamin E.J., Chyou J.Y., Cronin E.M., Deswal A., Eckhardt L.L., Goldberger Z.D., Gopinathannair R. (2024). 2023 ACC/AHA/ACCP/HRS Guideline for the Diagnosis and Management of Atrial Fibrillation: A Report of the American College of Cardiology/American Heart Association Joint Committee on Clinical Practice Guidelines. Circulation.

[8] Van Gelder I.C., Rienstra M., Bunting K.V., Casado-Arroyo R., Caso V., Crijns H.J.G.M., De Potter T.J.R., Dwight J., Guasti L., Hanke T. (2024). 2024 ESC Guidelines for the management of atrial fibrillation developed in collaboration with the European Association for Cardio-Thoracic Surgery (EACTS). Eur. Heart J..

[9] Verma A., Jiang C.-Y., Betts T.R., Chen J., Deisenhofer I., Mantovan R., Macle L., Morillo C.A., Haverkamp W., Weerasooriya R. (2015). Approaches to catheter ablation for persistent atrial fibrillation. N. Engl. J. Med..

[10] Kistler P.M., Chieng D., Sugumar H., Ling L.H., Segan L., Azzopardi S., Al-Kaisey A., Parameswaran R., Anderson R.D., Hawson J. (2023). Effect of Catheter Ablation Using Pulmonary Vein Isolation with vs Without Posterior Left Atrial Wall Isolation on Atrial Arrhythmia Recurrence in Patients with Persistent Atrial Fibrillation: The CAPLA Randomized Clinical Trial. JAMA.

[11] Goette A., Kalman J.M., Aguinaga L., Akar J., Cabrera J.A., Chen S.A., Chugh S.S., Corradi D., D’Avila A., Dobrev D. (2016). EHRA/HRS/APHRS/SOLAECE expert consensus on atrial cardiomyopathies: Definition, characterization, and clinical implication. Europace.

[12] Wijesurendra R.S., Casadei B. (2019). Mechanisms of atrial fibrillation. Heart.

[13] Nattel S., Dobrev D. (2012). The multidimensional role of calcium in atrial fibrillation pathophysiology: Mechanistic insights and therapeutic opportunities. Eur. Heart J..

[14] Schotten U., Verheule S., Kirchhof P., Goette A. (2011). Pathophysiological mechanisms of atrial fibrillation: A translational appraisal. Physiol. Rev..

[15] Irie T., Yu R., Bradfield J.S., Vaseghi M., Buch E.F., Ajijola O., Macias C., Fujimura O., Mandapati R., Boyle N.G. (2015). Relationship between sinus rhythm late activation zones and critical sites for scar-related ventricular tachycardia: Systematic analysis of isochronal late activation mapping. Circ. Arrhythm. Electrophysiol..

[16] Jia H., Wang W., Yu B. (2023). Efficacy and safety of low voltage area ablation for atrial fibrillation: A systematic review and meta-analysis. J. Interv. Card. Electrophysiol..

[17] Mitran R.-E., Popa-Fotea N.-M., Iorgulescu C., Nastasa A., Pupaza A., Gondos V., Petre I.-G., Paja S.-C., Vatasescu R.-G. (2023). Left Atrial Low-Voltage Areas Predict the Risk of Atrial Fibrillation Recurrence after Radiofrequency Ablation. Biomedicines.

[18] Oakes R.S., Badger T.J., Kholmovski E.G., Akoum N., Burgon N.S., Fish E.N., Blauer J.J., Rao S.N., DiBella E.V., Segerson N.M. (2009). Detection and quantification of left atrial structural remodeling with delayed-enhancement magnetic resonance imaging in patients with atrial fibrillation. Circulation.

[19] Nery P.B., Alqarawi W., Nair G.M., Sadek M.M., Redpath C.J., Golian M., Al Dawood W., Chen L., Hansom S.P., Klein A. (2020). Catheter Ablation of Low-Voltage Areas for Persistent Atrial Fibrillation: Procedural Outcomes Using High-Density Voltage Mapping. Can. J. Cardiol..

[20] Ammar-Busch S., Buiatti A., Tatzber A., Reents T., Bourier F., Semmler V., Telishevska M., Hessling G., Deisenhofer I. (2020). Predictors of low voltage areas in persistent atrial fibrillation: Is it really a matter of time?. J. Interv. Card. Electrophysiol..

[21] Cameli M., Mandoli G.E., Loiacono F., Dini F.L., Henein M., Mondillo S. (2016). Left atrial strain: A new parameter for assessment of left ventricular filling pressure. Heart Fail Rev..

[22] Yuda S. (2021). Current clinical applications of speckle tracking echocardiography for assessment of left atrial function. J. Echocardiogr..

[23] Pathan F., D’ELia N., Nolan M.T., Marwick T.H., Negishi K. (2017). Normal Ranges of Left Atrial Strain by Speckle-Tracking Echocardiography: A Systematic Review and Meta-Analysis. J. Am. Soc. Echocardiogr..

[24] Cameli M., Mandoli G.E., Loiacono F., Sparla S., Iardino E., Mondillo S. (2016). Left atrial strain: A useful index in atrial fibrillation. Int. J. Cardiol..

[25] Kiliszek M., Uziębło-Życzkowska B., Krzyżanowski K., Jurek A., Wierzbowski R., Smalc-Stasiak M., Krzesiński P. (2023). Value of Left Atrial Strain in Predicting Recurrence after Atrial Fibrillation Ablation. J. Clin. Med..

[26] Ma J., Chen Q., Ma S. (2021). Left atrial fibrosis in atrial fibrillation: Mechanisms, clinical evaluation and management. J. Cell. Mol. Med..

[27] Calkins H., Reynolds M.R., Spector P., Sondhi M., Xu Y., Martin A., Williams C.J., Sledge I. (2009). Treatment of atrial fibrillation with antiarrhythmic drugs or radiofrequency ablation: Two systematic literature reviews and meta-analyses. Circ. Arrhythm. Electrophysiol..

[28] Andrade J.G., Champagne J., Dubuc M., Deyell M.W., Verma A., Macle L., Leong-Sit P., Novak P., Badra-Verdu M., Sapp J. (2019). Cryoballoon or Radiofrequency Ablation for Atrial Fibrillation Assessed by Continuous Monitoring: A Randomized Clinical Trial. Circulation.

[29] Mörtsell D., Arbelo E., Dagres N., Brugada J., Laroche C., Trines S.A., Malmborg H., Höglund N., Tavazzi L., Pokushalov E. (2019). Cryoballoon vs. radiofrequency ablation for atrial fibrillation: A study of outcome and safety based on the ESC-EHRA atrial fibrillation ablation long-term registry and the Swedish catheter ablation registry. Europace.

[30] Mălăescu G.-G., Mirea O., Capotă R., Petrescu A.M., Duchenne J., Voigt J.-U. (2022). Left Atrial Strain Determinants During the Cardiac Phases. JACC Cardiovasc. Imaging.

[31] Nishida K., Qi X.Y., Wakili R., Comtois P., Chartier D., Harada M., Iwasaki Y.-K., Romeo P., Maguy A., Dobrev D. (2011). Mechanisms of atrial tachyarrhythmias associated with coronary artery occlusion in a chronic canine model. Circulation.

[32] Reant P., Lafitte S., Jaïs P., Serri K., Weerasooriya R., Hocini M., Pillois X., Clementy J., Haïssaguerre M., Roudaut R. (2005). Reverse remodeling of the left cardiac chambers after catheter ablation after 1 year in a series of patients with isolated atrial fibrillation. Circulation.

[33] Khan H.R., Yakupoglu H.Y., Kralj-Hans I., Haldar S., Bahrami T., Clague J., De Souza A., Hussain W., Jarman J., Jones D.G. (2023). Left Atrial Function Predicts Atrial Arrhythmia Recurrence Following Ablation of Long-Standing Persistent Atrial Fibrillation. Circ. Cardiovasc. Imaging.

[34] Kuppahally S.S., Akoum N., Burgon N.S., Badger T.J., Kholmovski E.G., Vijayakumar S., Rao S.N., Blauer J., Fish E.N., Dibella E.V. (2010). Left atrial strain and strain rate in patients with paroxysmal and persistent atrial fibrillation: Relationship to left atrial structural remodeling detected by delayed-enhancement MRI. Circ. Cardiovasc. Imaging.

[35] Laish-Farkash A., Brezinov O.P., Valdman A., Tam D., Rahkovich M., Kogan Y., Marincheva G. (2021). Evaluation of left atrial remodeling by 2D-speckle-tracking echocardiography versus by high-density voltage mapping in patients with atrial fibrillation. J. Cardiovasc. Electrophysiol..

[36] Rossi M., Bucci T., Tartaglia E., Zhao M., Lam S.H.M., Chen Y., Askarinejad A., Liu H., Ferri C., Boriani G. (2026). Clinical characteristics and outcomes of first diagnosed atrial fibrillation: Insights from 2 prospective registries in Europe and Asia. Heart Rhythm..

[37] Sugimoto T., Robinet S., Dulgheru R., Bernard A., Ilardi F., Contu L., Addetia K., Caballero L., Kacharava G., Athanassopoulos G.D. (2018). Echocardiographic reference ranges for normal left atrial function parameters: Results from the EACVI NORRE study. Eur. Heart J. Cardiovasc. Imaging.

[38] Masuda M., Sunaga A., Tanaka N., Watanabe T., Minamiguchi H., Egami Y., Oka T., Minamisaka T., Kanda T., Okada M. (2025). Low-voltage-area ablation for persistent atrial fibrillation: A randomized controlled trial. Nat. Med..

[39] Zhou Y., Zhang H., Yan P., Zhou P., Wang P., Li X. (2022). Efficacy of left atrial low-voltage area-guided catheter ablation of atrial fibrillation: An updated systematic review and meta-analysis. Front. Cardiovasc. Med..

[40] Yamaguchi T., Otsubo T., Takahashi Y., Nakashima K., Fukui A., Hirota K., Ishii Y., Shinzato K., Osako R., Tahara M. (2022). Atrial Structural Remodeling in Patients with Atrial Fibrillation Is a Diffuse Fibrotic Process: Evidence from High-Density Voltage Mapping and Atrial Biopsy. J. Am. Heart Assoc..

[41] Zhang R., Li H., Wang Y., Yu T., Li J., Wu Y., Yu Z., Liang C., Yu D., Xue L. (2025). Left atrial strain predicts paroxysmal atrial fibrillation recurrence after catheter ablation: A 1-year study using three-dimensional speckle-tracking echocardiography. BMC Cardiovasc. Disord..

[42] Li Y., Li Y., Sun L., Ye X., Cai Q., Zhu W., Guo D., Ding X., Wang J., Lv X. (2022). Left atrial strain for predicting recurrence in patients with non-valvular atrial fibrillation after catheter ablation: A single-center two-dimensional speckle tracking retrospective study. BMC Cardiovasc. Disord..

[43] Chang S., Zhang X., Ge C., Zhong Y., Zeng D., Cai Y., Huang T., Wu J. (2024). Automatic Echocardiographic Assessment of Left Atrial Function for Prediction of Low-Voltage Areas in Non-Valvular Atrial Fibrillation. Int. J. Gen. Med..

