# Supplementary material for: Predictive Value of Left Atrial Strain Combined with Low-Voltage Substrate for Recurrence After Radiofrequency Catheter Ablation in Persistent Atrial Fibrillation
Source: Diagnostics (Basel). 2026 Oct 11;16(20):3303. doi: 10.3390/diagnostics16203303 (PMC13703644; doi:10.3390/diagnostics16203303)
Supplement: Supplementary file 1 [file diagnostics-16-03303-s001.zip › diagnostics-4558730-supplementary.pdf]

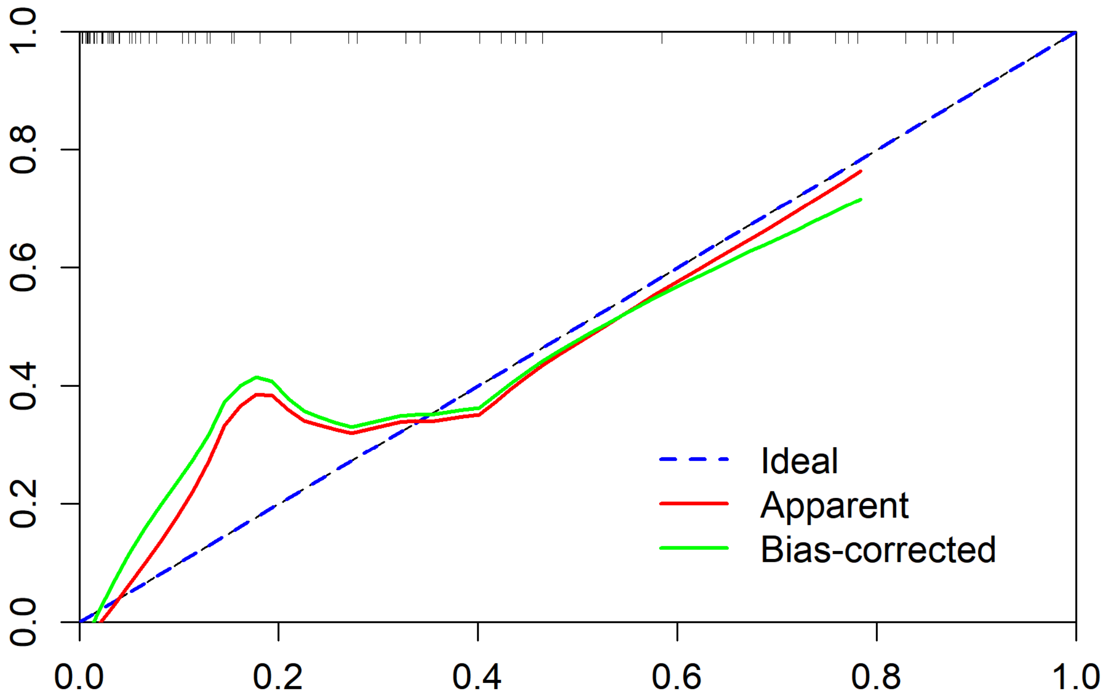

Figure S1. Calibration curve for Model 1. The blue dashed line represents the ideal calibration (45-degree reference line). The red solid line represents the apparent calibration. The green solid line represents the bias-corrected calibration via 1000 Bootstrap resamples.

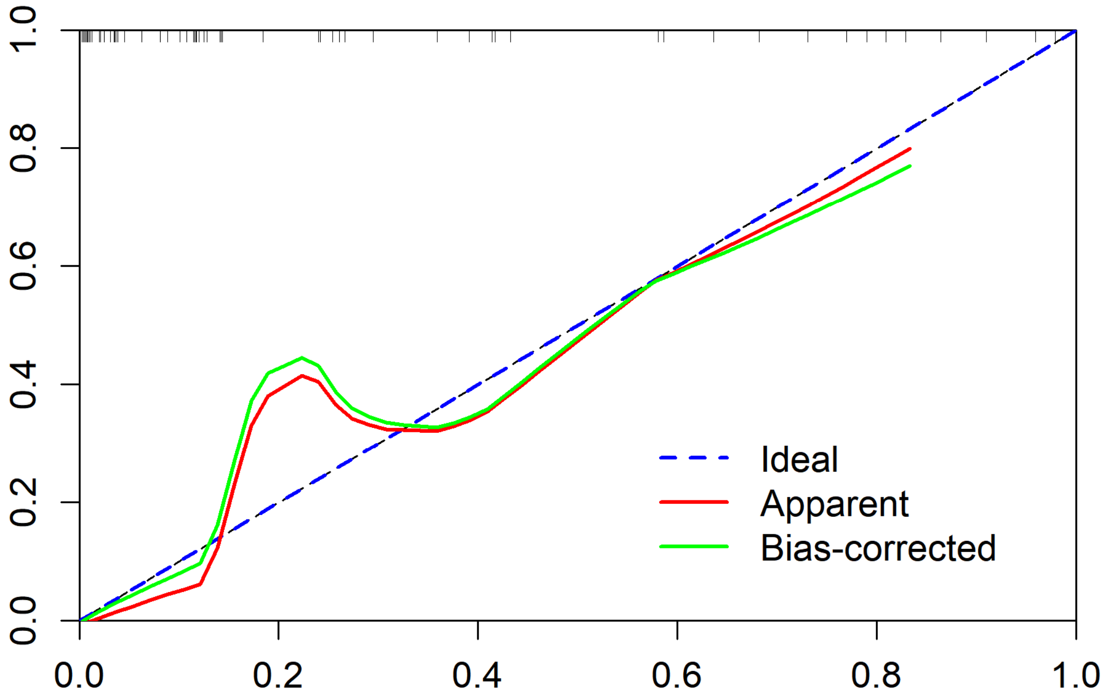

Figure S2. Calibration curve for Model 2. The blue dashed line represents the ideal calibration (45-degree reference line). The red solid line represents the apparent calibration. The green solid line represents the bias-corrected calibration via 1000 Bootstrap resamples.
